# Effect of Lap Joint Configuration and Seam Strategy in Green-Laser Welding on Multi-Layer Cu Foil Stacks to Lead-Tab Joints for Pouch Cell Application

**DOI:** 10.3390/ma19030573

**Published:** 2026-02-02

**Authors:** Seong Min Hong, Bum-Su Go, Hee-Seon Bang

**Affiliations:** 1 Joining and Welding Research Institute, The University of Osaka, Osaka 567-0047, Japan; hong.sm.jwri@osaka-u.ac.jp; 2 Research Institute, Korea Welding and Joining Engineering Association, Gwangju 61012, Republic of Korea; 3 Department of Welding and Joining Science Engineering, Chosun University, Gwangju 61452, Republic of Korea

**Keywords:** green-laser welding, copper foil stack, lead-tab joining, beam oscillation, electrical resistance, lithium-ion pouch cells

## Abstract

This study examines the joining characteristics of Cu foil stacks to lead tabs using green-laser welding in the main-welding step of a sequential welding process for lithium-ion pouch cells. The influence of lap configuration, line and wobble seam strategies, and process parameters was systematically investigated in terms of bead morphology, mechanical performance, metallurgical characteristics, and electrical resistance. Under the present line-welding parameter window (2.0 kW, 100–200 mm/s), humping, pinholes, and porosity were observed, particularly in the upper lead-tab configuration, which is attributed to melt-pool/keyhole instability under the applied conditions. Wobble welding effectively suppressed these defects in the foil-stack configuration by promoting stable melt flow and efficient bubble expulsion. Mechanical tests revealed that the wobble-based seam strategy achieved a maximum tensile–shear load of approximately 1.28 kN at a wobble amplitude of 0.8 mm. Fracture analysis confirmed a transition from seam-type interfacial failure in line welding to ductile tearing in the heat-affected zone with wobble welding. In electrical performance, wobble welding reduced resistance to as low as 45 µΩ at a wobble amplitude of 1.2 mm, while line welding yielded higher and scattered values. These results should be interpreted as the combined outcome of the wobble-based seam strategy (beam oscillation together with overlapped stitch welding at a lower travel speed) under the present processing windows. A strictly matched A/B comparison at identical linear energy density and seam layout will be investigated in future work to isolate the effect of oscillation.

## 1. Introduction

The demand for high-performance lithium-ion batteries (LIBs) has significantly increased due to the rapid growth of electric vehicles (EVs) and energy storage systems. In these batteries, the joining of Cu foil stacks to lead tabs is a critical process that determines the overall electrical efficiency and mechanical durability of the cells [1,2,3]. However, this joining step remains technically challenging because of the micro-scale thickness of the foils, multiple interfaces, and the potential for welding defects.

Ultrasonic welding has been widely applied for tab welding in LIBs because it is a solid-state process, which is capable of producing joints without significant thermal effects. However, several studies have reported its inherent limitations. For example, Shin et al. investigated ultrasonic welding of 40-layer Cu foil stacks to Ni-plated Cu strips and reported issues such as cracks, tearing, and plastic deformation at high clamping pressures [2]. Similarly, Arimoto et al. demonstrated that conventional knurled tools cause foil damage, whereas cylindrical tools alleviate this but with reduced strength [3]. Zhao et al. also confirmed that ultrasonic welding suffers from delamination and low peel strength due to uneven stress distribution [4]. To address these issues, advanced complex vibration systems with two-dimensional oscillation have been developed, requiring lower clamping pressure and improving joint quality compared to conventional ultrasonic welding [5,6].

Laser welding has emerged as an alternative due to its high energy density, precision, and ability to form deep penetration welds in a short time. A comprehensive review by Yang et al. [7] synthesized the application and development of blue and green lasers in industrial manufacturing, highlighting that short-wavelength lasers fundamentally alter the absorptivity of highly reflective metals and enable more stable process windows for dissimilar metal welding. The review noted that while green/blue lasers reduce required heat input and improve initial energy coupling, careful control of IMC formation and melt-pool dynamics remains essential to achieving joints with optimal mechanical strength and electrical conductivity. Nevertheless, copper’s low infrared (IR) absorption (<5%) and high thermal conductivity often result in unstable keyhole formation, spatter, and porosity [8,9].

Recent advances demonstrated that green-laser welding (515–532 nm) increases the absolute absorptivity of copper from a very low level in the near-IR (<5%) to the order of ~40–50% at room temperature (depending on surface condition), thereby improving the reproducibility of initial energy coupling and keyhole formation [10,11,12,13]. Grabmann et al. [14] employed a high-power green disk laser to join current collector foil stacks and established parameter windows that balance mechanical strength requirements with production throughput. Their statistical analysis of tensile–shear forces across different power-speed combinations confirmed that green-laser welding can achieve mechanically robust joints in thin-foil configurations while maintaining the electrical integrity necessary for internal battery contacting. Kaufmann et al. [15] investigated tailored laser beam shapes for copper welding using green radiation up to 3 kW and found that beam shaping modifies heat conduction regimes, leading to reduced spatter, improved seam regularity, and more consistent mechanical behavior. These improvements in seam quality directly translate to enhanced electrical performance through larger and more uniform current-carrying cross-sections. Jia et al. [16] provided mechanistic insights into absorptivity enhancement during continuous-wave green-laser welding of copper, identifying that redeposited nanoparticles (predominantly Cu_2_O) from the vapor plume increase surface absorptivity during processing, thereby enabling energy-efficient welding at lower power thresholds while producing smooth, continuous conductive tracks. Importantly, existing studies also report that improved coupling does not automatically eliminate defects, because porosity/humping formation remains governed by melt-pool/keyhole dynamics and the applied seam strategy (e.g., oscillation/beam shaping) [8,9,10,11]. For instance, Kamat et al. applied green-laser welding to foil-to-tab joints and achieved tensile–shear loads exceeding 800 N under optimal conditions [17], while Grabmann et al. reported enhanced seam strength in multi-layer Al and Cu foils [14]. However, challenges remain: Zhang et al. [9] and Li et al. [10] observed porosity and humping phenomena in high-power laser welding, which degrade strength and reliability.

Furthermore, studies such as those by Kumar et al. [11] and Dimatteo et al. [6] highlighted that oscillating laser beams (wobble welding) effectively suppress humping and porosity by stabilizing the melt pool. Similarly, Yoon and Bang [12] confirmed that beam wobbling reduces defects in Al/Cu joints, improving seam uniformity. Despite these advances, the combined effects of lap configuration and oscillation parameters on the joining of Cu foil stacks to lead tabs remain insufficiently investigated, particularly when joint integrity must be assessed simultaneously by mechanical strength and micro-ohm-level electrical resistance.

In the context of copper laser welding, the literature consistently reports that visible (green) wavelengths provide a markedly higher absolute absorptivity on copper (≈40–50% at room temperature) and thus improve the reproducibility of energy coupling at process start, although porosity/spatter can still occur depending on keyhole dynamics and ambient conditions. For multi-layer foil stacks, process strategies such as oscillation/beam shaping have been explored to broaden the feasible window and stabilize seam formation, but a systematic comparison between lap configuration and seam strategy under a sequential battery-tab joining context remains limited.

Therefore, the objective of this study is to systematically examine the joining characteristics of a 60-layer Cu foil stack to a Cu lead tab using green-laser welding in the main-welding step. By analyzing the effects of lap configuration and beam oscillation on weld morphology, mechanical strength, electrical resistance, and metallurgical evolution, this work seeks to establish optimized process conditions for achieving defect-free, high-performance joints in advanced LIB pouch cells.

## 2. Research Methods

### 2.1. Used Materials and Welding Conditions

**Figure 1:**
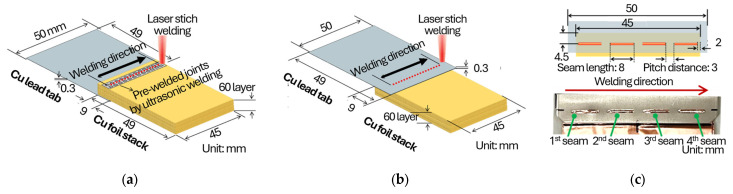
Schematic illustration of lap configuration in laser welding. (**a**) Upper foil-stack configuration. (**b**) Upper lead-tab configuration. (**c**) Details of laser stitch welding.

The joining experiments were performed on a foil stack consisting of sixty layers of battery-grade bare copper foil (Cu-PHC, 8 µm thickness). To reduce macroscopic interfacial separation and improve handling stability prior to laser welding, the stack was pre-welded using ultrasonic welding with a clamping pressure of 1.0 bar, a vibrational amplitude of 25 µm, and a welding energy of 600 J. In the present study, ultrasonic welding was employed as a preprocessing step to consolidate the 60-layer foil stack prior to laser welding. The primary role of this step is not to create a final metallurgical joint, but to densify the stack and suppress macroscopic interfacial separation between individual foils, thereby increasing the effective thickness and mechanical integrity of the stack during subsequent laser processing. From a laser-welding perspective, this consolidation improves handling stability, reduces foil fluttering or local separation under recoil pressure, and provides a more continuous thermal and mechanical boundary condition for melt-pool formation. Without such preprocessing, individual thin foils can locally separate or vibrate during laser irradiation, which would exacerbate melt-pool instability, promote irregular keyhole behavior, and increase the likelihood of spatter and gas entrapment. Therefore, ultrasonic pre-welding facilitates more reproducible green-laser coupling and melt-pool behavior by transforming the foil stack into a quasi-monolithic target with increased effective thickness. This approach also reflects practical joining scenarios in pouch-cell manufacturing, where joining is typically performed between pre-assembled components rather than between freely stacked individual foils. The lead-tab material was commercial pure copper (C1020, 0.3 mm thickness) with a nickel coating of approximately 10 µm on both sides. Note that this pre-welding can still leave indentation–imprinted micro-voids/micro-gaps at local interfaces, which may serve as preferential sites for pinhole initiation during subsequent laser welding. As shown in Figure 1, both foils and tabs were prepared in coupon form, with dimensions of 49 mm × 45 mm and 49 mm × 50 mm, respectively. Two lap configurations were adopted: one with the foil stack placed on top of the lead tab, and another with the lead tab placed on top of the foil stack, each with an overlap length of 9 mm. Moreover, the weld locations of the overlap area were carried out using laser-stitch welding, with a seam length of 8 mm and a pitch distance of 3 mm to prevent excessive welding deformation and burn through [5], including line and oscillation strategies as shown Figure 1c. Figure 1c shows an optical image of the actual laser welding setup, from which the beam travel direction, wobble path orientation, and focal position can be directly identified. The laser beam travels along the longitudinal direction of the overlap region shown in the lap configuration. For wobble welding, the oscillation is applied perpendicular to the beam travel direction within the overlap area. The focal position is set at the upper surface of the foil stack, ensuring interaction with the multiple foil interfaces beneath during welding. The schematic is intended to illustrate the relative arrangement of the Cu foil stack and the Ni-coated Cu lead tab for the two lap configurations. In the actual experiments, the laser beam was scanned along the overlap region indicated in the schematic, following the seam direction parallel to the long axis of the joint. For wobble welding, the beam oscillation was superimposed onto this primary scan path, whereas for line welding, a straight-line scan without oscillation was applied. The schematic therefore represents the geometric relationship between the lap configuration, joint interface, and laser scanning strategy, rather than an exact scale drawing of the weld bead geometry.

The main welding process was conducted using a disk laser system (TruDisk 3022, TRUMPF GmbH, Ditzingen, Germany) with a maximum output power of 3 kW at a wavelength of 515 nm. A 2-in-1 optical fiber based on Bright Line Weld (BLW) technology was employed to stabilize the keyhole and reduce spatter. Two welding strategies were examined. In the line welding condition, the laser power was maintained at 2 kW, while the welding speed was varied between 100 and 200 mm/s. The ring-core power ratio was fixed at 50%. In the oscillation welding condition, the laser power, welding speed, and overlap rate were fixed at 2 kW, 50 mm/s, and 80%, respectively, while the wobble amplitude was varied from 0.4 to 1.2 mm, corresponding to wobble frequencies of 625, 313, and 209 Hz. A stitch welding approach was applied with a seam length of 8 mm, a pitch of 3 mm, and four seams per overlap. Nitrogen shielding gas was supplied at 20 L/min to mitigate porosity formation, as nitrogen shielding has been reported to reduce porosity in laser welding (particularly in Al alloys) by influencing keyhole/bubble behavior and gas entrapment, while maintaining practical cost and availability for high-throughput manufacturing [18,19]. Summarized welding parameters for the above are organized in Table 1, and chemical composition and mechanical properties of the material used in this study are shown in Table 2.

### 2.2. Evaluation of the Weldability of the Foil Stack-to-Lead Tab Joints

The ultrasonic pre-welding step locally compacted the foil stack and produced shallow indentation marks on the stack surface; these indentation-related micro-gaps can act as preferential sites for pinhole initiation during subsequent laser welding. The welded joints were evaluated in terms of bead morphology, mechanical strength, microstructure, and electrical performance. Bead morphology was observed using an optical macroscope, and defects such as porosity, humping, underfill, and burn-through were identified. The bead width was measured at both the top and bottom surfaces. Mechanical properties were assessed through tensile–shear testing conducted on a universal testing machine (Galdabini Quasar 5, 5 kN capacity) at a crosshead speed of 10 mm/min. To ensure accurate measurement, the width of the lead tab was machined to match the foil stack, and the clamping jaws were offset to reduce bending effects. The maximum load at fracture was recorded as the joint strength, and each condition was tested three times for reproducibility. For hardness evaluation, Vickers microhardness measurements were performed across the weld cross-sections using a load of 100 g, a dwell time of 10 s, and a spacing of 0.1 mm. The metallurgical characteristics of the joints were examined by polishing and etching the cross-sections with an ethanol–HCl–FeCl_3_ solution, followed by optical microscopy and scanning electron equipped with energy-dispersive spectroscopy (EDS). Electrical performance was investigated by measuring the contact resistance of the welded joints with a four-point probe method (RM3548, Hioki, Nagano, Japan). A constant current of 1.0 A was applied at room temperature, and the resistance value was recorded after 60 s to ensure stable readings. Measurement geometry and uncertainty were assessed by calculating average and standard deviation. The reported value represents the joint-level contact resistance measured using a Kelvin configuration, where the current was injected through the outer probes and the voltage was sensed by the inner probes. The probe spacing (inner voltage–probe distance) was fixed, and the measurement span was centered on the welded overlap so that the dominant potential drop traversed the welded interface. Because the total resistance is in the micro-ohm range, all measurements were performed with the same probe fixture, probe materials, and contact pressure to minimize fixture-dependent scatter. The same current path length and specimen clamping condition were maintained across all tests; therefore, while the measured value inherently includes small bulk contributions from the tab/foil within the gauge length, the between-condition comparison remains valid because the geometry was identical. Temperature sensitivity was minimized by conducting all measurements at room temperature and recording values after a 60 s stabilization period.

## 3. Results and Discussion

### 3.1. Bead Profiles

**Figure 2:**
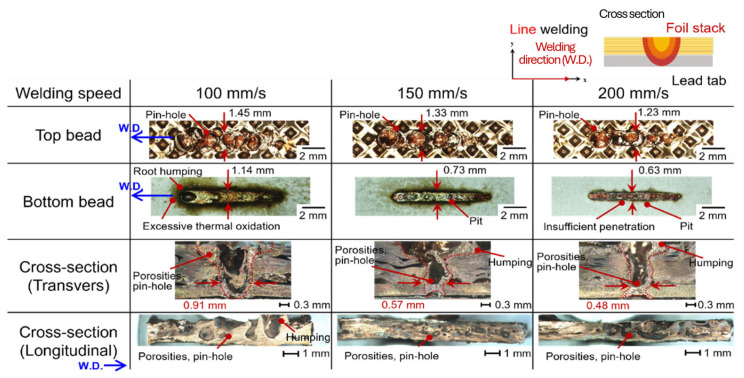
Schematic illustration of the welding path with the experimental set-up of the upper foil-stack configuration line welding and the corresponding bead profiles with various welding speeds.

**Figure 3:**
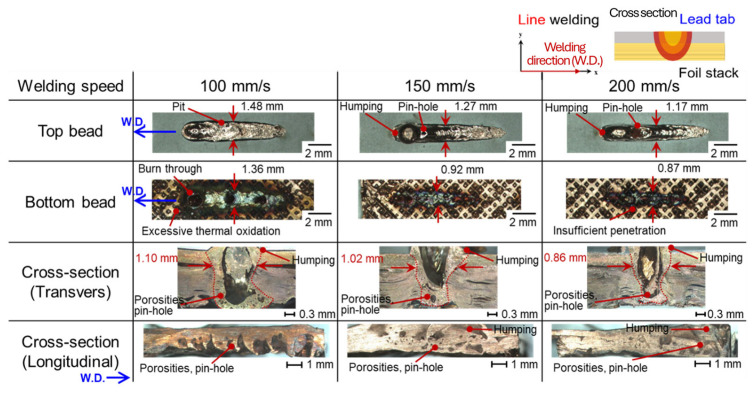
Schematic illustration of the welding path with the experimental set-up of the upper lead-tab configuration line welding and the corresponding bead profile with various welding speeds.

Bead profile characteristics were investigated according to lap configuration, seam strategies (line and wobble), and process parameters. Figure 2 shows the bead profiles at a laser power of 2.0 kW with welding speeds ranging from 100 to 200 mm/s for the upper foil-stack configuration in line welding. Pinhole defects were consistently observed on the top surface regardless of welding speed, which is attributed to micro-gaps formed between indentations in the foil stack. Figure 3 shows the bead appearance under identical conditions for the upper lead-tab configuration. Humping and pinhole defects were present across all welding speeds, caused by the internal expansion force exceeding the surface tension of the molten pool, leading to unstable bead formation.

**Figure 4:**
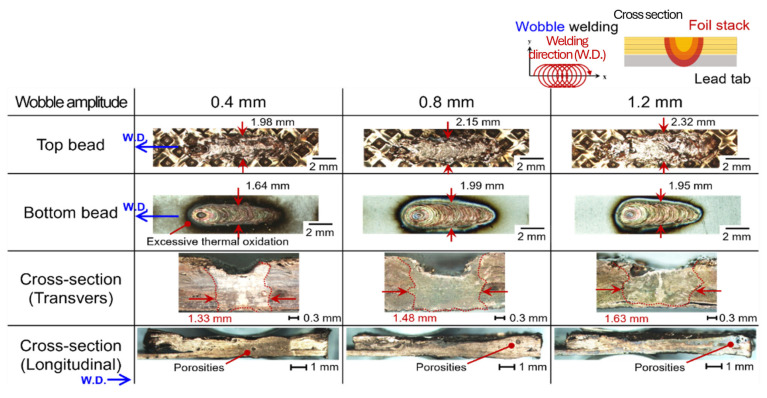
Schematic illustration of the welding path with the experimental set-up of the upper foil-stack configuration wobble welding and the corresponding bead profile with various wobble amplitudes.

**Figure 5:**
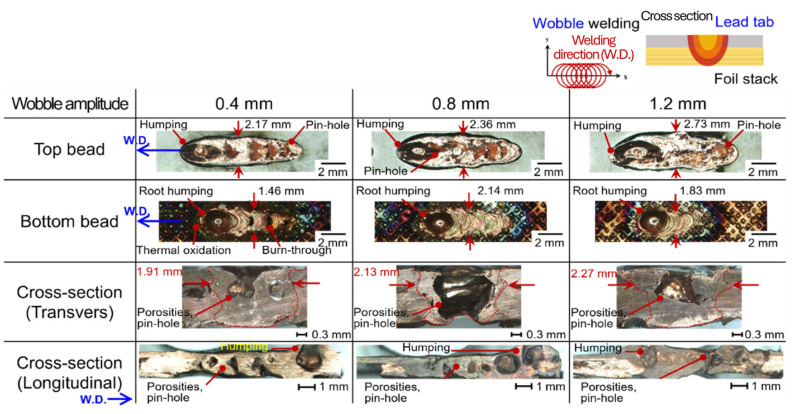
Schematic illustration of the welding path with the experimental set-up of the upper lead-tab configuration wobble welding and the corresponding bead profile with various wobble amplitudes.

Figure 4 shows the effect of wobble amplitudes ranging from 0.4 mm to 1.2 mm in the upper foil-stack configuration. In this case, humping and pinhole defects were effectively suppressed. The combination of circular and linear oscillation stabilized melt pool dynamics, facilitating efficient expulsion of vapor and bubbles, and resulting in a sound welded joint. Figure 5 shows the bead appearance of the upper lead-tab configuration under wobble welding. Despite variation in wobble amplitude, humping and pinhole defects persisted. Although the present dataset does not include time-resolved diagnostics (e.g., high-speed imaging) or thermal simulations, the persistence of humping/pinhole defects in the upper lead-tab configuration is most consistently interpreted as a configuration-driven difference in thermal boundary conditions and interfacial support, rather than as an intrinsic difference in the thermal conductivity of the joining partners. Specifically, the lead tab is a monolithic Cu sheet (0.3 mm) with a Ni coating (~10 µm), which provides an efficient heat-extraction pathway into the surrounding bulk and fixturing. This is expected to shorten the molten-pool lifetime and narrow the process tolerance against hydrodynamic instabilities and keyhole collapse, thereby making defect suppression by wobble-assisted melt agitation less effective than in the foil-stack-top configuration. In contrast, the foil stack introduces multiple interfaces and effective thermal contact resistances, which can reduce lateral heat spreading and, under the wobble-based seam strategy, promote a wider remelted/bonded interface with fewer discontinuities. This configuration-dependent sensitivity is consistent with prior copper green-laser studies showing that defect formation remains strongly coupled to local heat balance and keyhole stability, even under improved absorption. Therefore, seam strategies that stabilize melt flow can reduce defect occurrence, but their effectiveness can still differ depending on thermal boundary conditions and interfacial support. A strictly validated mechanism will require coupled thermal/flow analysis or time-resolved observations and is therefore left for future work.

Although the present study does not include time-resolved thermal diagnostics or numerical heat-transfer simulations, an order-of-magnitude heat-balance argument supports why the upper lead-tab configuration is more prone to melt-pool instability. The lead tab is a monolithic Cu sheet (0.3 mm) that provides a continuous heat sink to the bulk and the fixturing. Using typical copper properties (ρ ≈ 8.96 g/cm^3^, cp ≈ 385 J/kg·K), the areal heat capacity of the lead tab is on the order of ρ·cp·t ≈ 1.0 × 10^3^ J/m^2^·K, meaning that a given local heat input produces a smaller temperature rise and a shorter molten lifetime when the heat extraction path is efficient. In addition, the characteristic thermal diffusion time across the tab thickness can be estimated as t_d ≈ t^2^/α; with α ≈ 1 × 10^−4^ m^2^/s for copper, and t = 3 × 10^−4^ m, t_d is on the order of 10^−3^ s, indicating that heat can be rapidly conducted away through the tab thickness during welding.

By contrast, the foil-stack surface involves multiple interfaces and effective thermal contact resistances, which can reduce the effective through-thickness heat flow and prolong the local molten-pool lifetime. Under wobble welding, oscillatory remelting can widen the bonded interface; however, in the upper lead-tab configuration, the combination of rapid heat dissipation into the monolithic tab and additional convective losses to the surrounding gas (particularly for a thin, exposed top sheet) can limit the time available for keyhole stabilization and bubble escape, leading to poorer bead stability even under oscillation. Therefore, the present interpretation is that the configuration imposes a more stringent thermal boundary condition (heat-sink dominated regime), narrowing the stable process window relative to the foil-stack-top configuration.

To provide a minimal quantitative descriptor of defect robustness without additional experiments, the occurrence of visible pinholes/porosity and severe humping was screened across all stitched seams (four seams per specimen, *n* = 3 specimens per condition). The wobble-based seam strategy reduced the fraction of seams exhibiting visible pinholes/porosity compared with the line-welding window, which is consistent with the lower variability observed in the measured tensile–shear peak load and electrical resistance under the present *n* = 3 dataset.

### 3.2. Mechanical Performance

The tensile–shear load and corresponding failure modes were analyzed to evaluate the mechanical performance of foil stack-to-lead tab joints under different welding strategies. Before discussing the mechanical performance, defect robustness was screened by optical microscopy (OM), counting stitched seams that exhibited visible pinholes, porosity or severe humping (four seams per specimen, *n* = 3 specimens per condition). Defects were classified according to standard criteria: pinholes (through-thickness holes), porosity (surface gas cavities), and humping (melt-pool instability protrusions). This OM-based screening indicates that the wobble-based strategy reduces defect occurrence, which is consistent with the reduced scatter in tensile–shear peak load.

**Figure 6:**
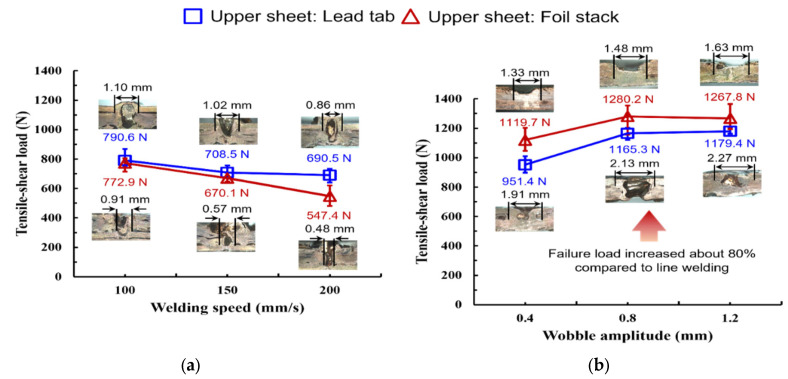
Tensile–shear peak load of joints produced by (**a**) line welding which is depending on the welding speed and (**b**) wobble welding which is depending on the wobble amplitude.

Figure 6 shows the comparison of tensile–shear load between line- and wobble-welded joints. Figure 6a compares the tensile–shear peak load of line-welded joints at 2.0 kW with welding speeds of 100–200 mm/s. The peak load generally decreased with increasing welding speed in both lap configurations, indicating reduced effective bonding at higher speeds. Depending on the lap configuration, the maximum load at 100 mm/s was approximately 0.77–0.79 kN, which decreased to approximately 0.55–0.69 kN at 200 mm/s. This trend is consistent with reduced melt volume and a narrower effective conduction/bonding width at higher welding speeds. All tensile–shear results are reported as mean ± 10 (*n* = 3) for each condition (see Figure 6). Although a jaw-offset fixture and width-matched tabs were used to reduce secondary bending, some residual mixed-mode loading may still remain due to the thin, compliant foil stack. To check for gross bending artifacts, the load–displacement compliance and fracture location were compared across repeats, and no anomalous outliers indicating dominant bending-driven failure were observed within the present dataset. A full-field strain measurement (e.g., DIC) will be considered in future work for a more rigorous separation of mode–mixity.

In contrast, the wobble-based seam strategy (50 mm/s with overlapped stitching) markedly increased the tensile–shear peak load and reduced scatter (Figure 6b). Across the two lap configurations, the peak load increased from approximately 0.95–1.12 kN at 0.4 mm amplitude to approximately 1.18–1.28 kN at 0.8–1.2 mm. The maximum load reached approximately 1.28 kN at a wobble amplitude of 0.8 mm, suggesting that an intermediate amplitude provides a favorable balance between interface widening and stable melt redistribution.

Furthermore, the mechanical response of line-welded joints can be consistently interpreted by the evolution of the effective bead (bonded-region) width with welding speed. As the welding speed increased, the bead width decreased from 1.10 mm to 0.86 mm in the upper lead-tab configuration and from 0.91 mm to 0.48 mm in the upper foil-stack configuration. This pronounced width reduction at higher speeds indicates a narrower effective bonding region due to the reduced linear energy density (P/v) and a smaller molten-pool footprint, which in turn limits both the load-transfer area under tensile–shear loading and the continuity of the metallurgical contact. Accordingly, the tensile–shear peak load decreased with welding speed in both lap configurations, with a more pronounced degradation trend in the upper foil-stack configuration, where the bonded width becomes particularly narrow at high speed. In contrast, the wobble-based seam strategy promoted a systematic widening of the bonded region with increasing wobble amplitude. In the upper lead-tab configuration, the bead width increased from 1.33 mm at an amplitude of 0.4 mm to 1.63 mm at 1.2 mm, while in the upper foil-stack configuration the bead width increased from 1.91 mm to 2.27 mm over the same amplitude range. The enlarged width is attributable to oscillation-assisted melt redistribution and an expanded effective interaction area, which together improve defect tolerance and enlarge the metallurgical contact region. This widening provides a physically consistent basis for the simultaneously improved tensile–shear performance and reduced electrical resistance under wobble welding: a wider bonded region increases the effective load-transfer area and mitigates current constriction at the joint, thereby lowering the micro-ohm-level resistance. Notably, the foil-stack configuration exhibited a larger bonded width throughout the wobble-amplitude window, indicating that the wobble strategy is more effective in stabilizing and widening the joint in the foil-stack configuration than in the upper lead-tab configuration under the present conditions.

When the tensile–shear results are considered together with the measured bead (bonded-region) widths; therefore, the mechanical trends can be interpreted in terms of the effective bonded footprint formed under each seam strategy and lap configuration. In line welding, increasing welding speed was accompanied by a reduction in bead width (upper lead-tab: 1.10 to 0.86 mm; upper foil-stack: 0.91 to 0.48 mm), which is consistent with the lower peak loads observed at higher speeds under the present parameter window. In wobble-based welding, increasing wobble amplitude produced a wider bonded region (upper lead-tab: 1.33 to 1.63 mm; upper foil-stack: 1.91 to 2.27 mm) and coincided with higher tensile–shear loads compared with the line condition. Although bead width alone does not fully determine joint strength, these observations suggest that the seam strategy and lap configuration primarily influence the tensile–shear performance by modifying the size and stability of the effective bonded region (i.e., the load-transfer area), rather than by a single factor in isolation.

**Figure 7:**
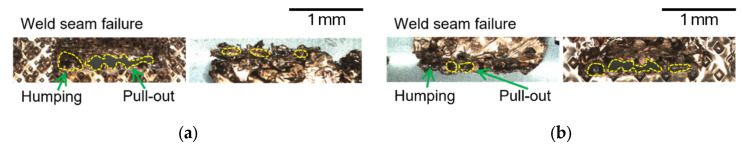
Comparison of the failure modes according to lap configuration in line welding. (**a**) Upper foil-stack configuration and (**b**) Upper lead-tab configuration.

The fracture surfaces of line-welded joints revealed seam-type failures dominated by interfacial cracks propagating along porosity-rich regions and incomplete fusion zones as shown in Figure 7a. In the lead-tab configuration, as shown in Figure 7b, partial penetration resulted in localized tearing and relatively low fracture loads, confirming the limited bonding effectiveness of line welding.

**Figure 8:**
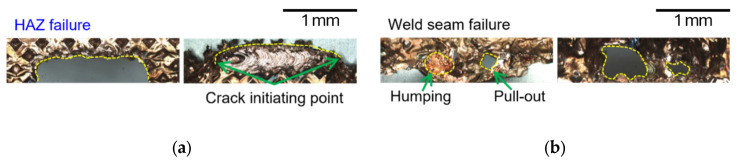
Comparison of failure modes according to lap configuration in wobble welding. (**a**) Upper foil-stack configuration and (**b**) Upper lead-tab configuration.

**Figure 9:**
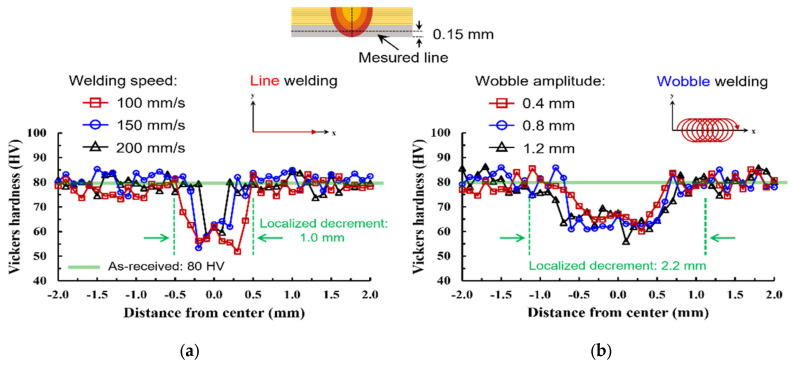
Comparison of hardness distribution between the line- and wobble-welded joint. (**a**) Line welding (without oscillation). (**b**) Wobble welding (with oscillation).

The fracture morphologies of wobble-welded joints confirmed the superior joint integrity. In the foil-stack configuration, ductile tearing occurred in the heat-affected zone (HAZ), indicating strong metallurgical bonding and refined microstructures, as shown in Figure 8a. In the lead-tab configuration, mixed-mode fractures were observed, but with higher loads and more uniform tearing compared to line welding, as shown in Figure 8b. Figure 9 shows comparison of hardness distribution between the line- and wobble-welded joint. The hardness value was decreased about 35% compared to base material. Wobble welding exhibited a wider localized decrement due to increase in melt pool area.

Overall, the mechanical evaluation demonstrates that line welding is prone to seam-type failure due to porosity and insufficient fusion, resulting in reduced load-bearing capacity. The present strength level (≈1.28 kN under the wobble-based strategy) is in line with prior green-laser foil-to-tab joining studies that reported high joint loads under optimized coupling and stabilization conditions, while also emphasizing that process strategy is critical for achieving reliable performance in highly conductive copper systems [16,17]. By contrast, wobble welding promoted wider interfaces and improved molten pool stability and defect suppression, leading to ductile failure modes and significantly higher tensile–shear loads. Hence, the mechanical evaluation indicates that the wobble-based seam strategy promotes a wider effective bonded region and improved defect tolerance compared to the single-pass line welds under the present parameter windows. Within the wobble strategy, the systematic dependence on wobble amplitude highlights beam oscillation as a governing factor for melt redistribution and joint integrity.

### 3.3. Metallurgical Characteristics

**Figure 10:**
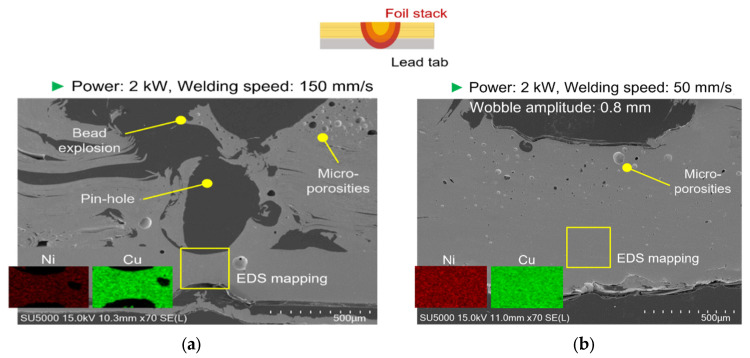
Comparison of metallographic with SEM-EDS between line- and wobble-welded joint. (**a**) Line welding. (**b**) Wobble welding.

Figure 10 compares the fusion-zone morphology and the elemental distribution (SEM-BSE/EDS) between representative line-welded and wobble-welded joints under the process windows defined in this study. In Figure 10a, the line-welded joint exhibits localized porosity and a less uniform bonding region, which is consistent with discontinuities in the effective conduction path. In contrast, in Figure 10b, the wobble-welded joint shows a more continuous remelted/bonded region with suppressed porosity and a more uniform fusion profile, which is in line with the improved mechanical integrity reported in Section 3.2. This shows that wobble welding produced a uniform fusion zone with refined grains and continuous elemental distribution, confirming improved metallurgical bonding. Furthermore, in line-welded joints (Figure 10a), localized porosities remained, while at wobble-welded joints (Figure 10b), a stable fusion zone with equiaxed grains was observed.

**Figure 11:**
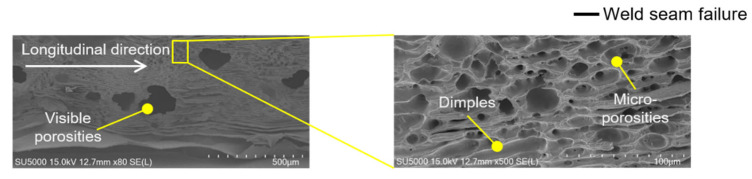
Fractured surface morphologies of the line-welded joint.

**Figure 12:**
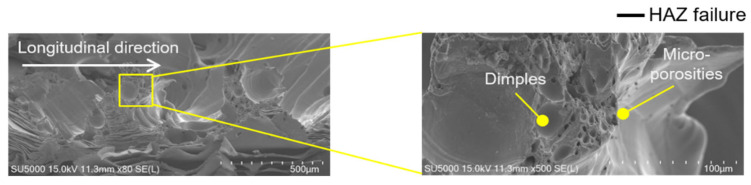
Fractured surface morphologies of the wobble-welded joint.

Figure 11 and Figure 12 present representative fracture-surface morphologies of the line-welded and wobble-welded joints, respectively, highlighting the transition from seam-type interfacial failure toward more ductile tearing when the bonding continuity and interface width are enhanced by the wobble-based seam strategy. In Figure 11, the line-welded joint (2.0 kW, 150 mm/s) exhibited porosities and bead explosion, with weak Ni response in the lead tab due to limited molten pool flow. In contrast, Figure 12 shows the wobble-welded joint (2.0 kW, 50 mm/s, 0.8 mm amplitude), which exhibited sound quality with only a few micro-porosities and stronger Ni distribution, indicating enhanced bonding.

Line welding (Figure 11) revealed porosities of 0.1–0.2 mm and micro-porosities coexisting with dimples, leading to seam-type failure. Wobble welding (Figure 12) exhibited only minor micro-porosities and ductile tearing in the HAZ, associated with higher fracture loads and improved reliability.

### 3.4. Electrical Resistance

**Figure 13:**
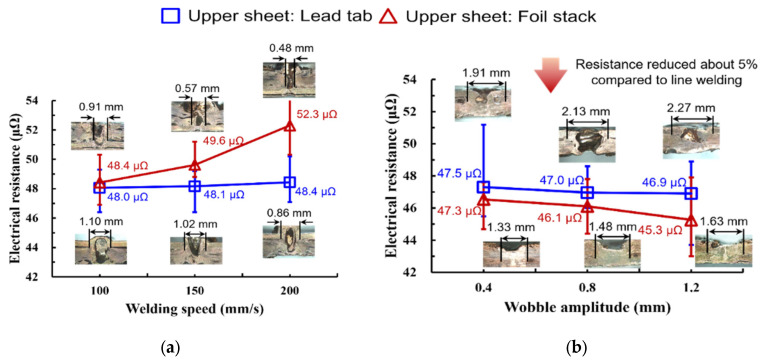
Comparison of electrical resistance between line- and wobble-welded joints as a function of welding speed (line) and wobble amplitude (wobble), evaluated for two lap configurations. (**a**) Electrical resistance of the joints in line welding depending on the welding speed. (**b**) Electrical resistance of the joints in wobble welding depending on the wobble amplitude.

Figure 13 compares the electrical resistance of line- and wobble-welded joints for two lap configurations (upper lead tab: blue squares; upper foil stack: red triangles). Under the present line-welding window (2.0 kW, 100–200 mm/s), the measured resistance remained in the micro-ohm range but exhibited configuration-dependent trends and scatter. For the upper lead-tab configuration, the mean resistance stayed nearly constant with welding speed (48.0 ± 0.37 µΩ at 100 mm/s, 48.1 ± 1.14 µΩ at 150 mm/s, and 48.4 ± 1.54 µΩ at 200 mm/s). In contrast, for the upper foil-stack configuration, the resistance tended to increase and become more scattered as the welding speed increased (48.4 ± 1.22 µΩ at 100 mm/s, 49.6 ± 1.22 µΩ at 150 mm/s, and 52.3 ± 1.79 µΩ at 200 mm/s). This behavior is consistent with discontinuities in the conduction path caused by process-induced defects, such as pores/pinholes, underfill, and locally incomplete bonding, which effectively reduce the conductive cross-section and introduce non-uniform current paths.

In contrast, the wobble-based seam strategy yielded lower and more stable resistance across both lap configurations. With increasing wobble amplitude (0.4–1.2 mm), the resistance decreased for the upper foil-stack configuration, reaching a minimum of 45.3 ± 0.69 µΩ at 1.2 mm (47.3 ± 0.46 µΩ at 0.4 mm; 46.1 ± 0.43 µΩ at 0.8 mm; 45.3 ± 0.69 µΩ at 1.2 mm). The upper lead-tab configuration also showed a smaller but consistent reduction (47.5 ± 0.86 µΩ at 0.4 mm; 47.0 ± 0.29 µΩ at 0.8 mm; 46.9 ± 0.28 µΩ at 1.2 mm), along with reduced scatter at higher amplitudes. The resistance reduction with increasing wobble amplitude can be rationalized by a constriction–resistance-dominated contact, where the effective metallic bonding width and continuity of the current path govern the measured value. Larger wobble amplitudes can widen the remelted/bonded interface and suppress discontinuities associated with pores or lack-of-fusion, thereby alleviating local current crowding and stabilizing the micro-ohm-level resistance. Because the absolute resistance is in the micro-ohm range, all measurements were performed using an identical four-point probe configuration and are reported.

In micro-ohm-level measurements, the dominant contribution arises from the constriction/contact resistance at the metallurgical bonding region, rather than from the bulk copper of the tab/foils. At smaller wobble amplitudes, the effective conduction path across the overlap is more discontinuous because the bonded region is narrower and locally interrupted by pores/underfill and incomplete bonding. In this case, the current is forced to pass through a reduced number of micro-contacts, leading to local current crowding and a higher apparent resistance.

In the present foil-stack joint, the electrical resistance is governed by the effective metallurgical bonding area across the overlap and its continuity, rather than by the bulk resistivity of copper. A wider bonded interface provides multiple parallel conduction pathways, whereas isolated pores, underfill, or local lack-of-fusion act as current constriction sites that locally increase resistance. Accordingly, even when the overall porosity level is qualitatively reduced, its spatial distribution along the bonding interface plays a critical role: clustered or interface-crossing pores are more detrimental to electrical conduction than dispersed, isolated pores.

With increasing wobble amplitude, the oscillatory remelting process promotes not only an expansion of the effective bonded width but also a redistribution and suppression of porosity at the interface, resulting in a more continuous metallurgical contact. This coupled evolution of bonding area and porosity distribution provides a mechanistic explanation for the observed reduction and stabilization of electrical resistance. Although the present study does not quantify bonding area or pore fraction explicitly, the consistent correlation between bead morphology, interfacial continuity, and resistance trends supports this interpretation.

As the wobble amplitude increases, the oscillatory remelting promotes (i) a wider effective bonded width and (ii) improved continuity of the bonded interface by reducing discontinuities associated with pores or lack-of-fusion. Consequently, the current path becomes less tortuous and more uniformly distributed across the overlap, alleviating current crowding and reducing constriction resistance. Beyond an optimum amplitude, however, excessive heat accumulation or unstable melt ejection could again introduce local discontinuities, which would counteract the benefit of interface widening. Therefore, the observed decrease and stabilization of resistance with wobble amplitude can be interpreted as a transition from a “discrete micro-contact dominated” interface to a “continuous metallurgical bonding dominated” interface within the overlap region.

### 3.5. Limitations and Scope of Comparison

It should be emphasized that the present comparison between line welding and wobble welding is not a strictly controlled A/B experiment in which only beam oscillation is varied. The wobble condition was implemented with a lower welding speed and an overlapped stitch layout, which inevitably modifies the effective energy input and the number of thermal cycles relative to a single-pass line weld. Therefore, the observed improvements should be interpreted as the combined effect of the wobble-based seam strategy (oscillation-assisted melt agitation together with overlapped stitching), rather than oscillation alone. Nevertheless, because all wobble trials were performed at fixed laser power, welding speed, overlap, and stitch geometry while varying only the wobble amplitude, the systematic trends with amplitude provide internal evidence that beam oscillation is a governing factor within the wobble strategy. A strictly matched comparison at identical linear energy density and identical seam layout will be addressed in future work. Notably, the nominal linear energy density (P/v) differs between the present windows: 10–20 J/mm for the line condition (2.0 kW, 100–200 mm/s) versus 40 J/mm for the wobble condition (2.0 kW, 50 mm/s), not accounting for the additional thermal cycling introduced by the 80% overlap and stitch layout.

In practical production, process selection is typically guided by meeting simultaneous acceptance criteria for both mechanical robustness and electrical resistance, rather than optimizing a single metric. In this context, the present results suggest that wobble amplitudes around 0.8–1.2 mm constitute a practical process window: 0.8 mm provides the highest tensile–shear load, whereas 1.2 mm yields the minimum resistance. A manufacturing-relevant implementation would therefore select an amplitude within this window based on the dominant requirement and process robustness, e.g., (i) choosing 1.0–1.2 mm when electrical efficiency (low resistance and its stability) is prioritized while maintaining sufficient mechanical strength, or (ii) choosing ~0.8–1.0 mm when mechanical robustness is prioritized while keeping resistance within specification. Notably, because the resistance differences are in the micro-ohm range and the strength differences may be more consequential for mechanical integrity; the final set-point should be determined by specification-driven trade-offs and robustness against variability (e.g., tolerance to fixture/contact conditions), rather than by the single best value of one metric.

From a manufacturing perspective, the proposed green-laser wobble welding process is inherently compatible with high-throughput battery production. The laser-based joining step itself operates on millisecond-to-subsecond time scales per seam, and the use of beam oscillation does not introduce a significant cycle-time penalty because it is implemented via scanner-based beam control rather than mechanical motion. Therefore, the overall cycle time is primarily governed by laser scanning length and stitch strategy, which are comparable to existing laser tab-welding operations.

Regarding alignment tolerance, wobble welding inherently relaxes positional sensitivity by distributing the energy over a finite oscillation width, making the process more tolerant to minor misalignment between the foil stack and the lead tab. This feature is particularly advantageous for stacked-foil assemblies, where small geometric variations are unavoidable in production. In terms of equipment complexity, the process relies on commercially available green-laser sources and standard galvanometer-based scanning optics. Apart from the laser wavelength, no fundamentally new hardware elements are required compared with conventional laser welding systems, suggesting that integration into existing battery production lines is feasible.

## 4. Conclusions

This study investigated the joining characteristics of Cu foil stacks to lead tabs using green-laser welding in the main-welding step of a sequential welding process. The influence of lap configuration, seam strategies (line and wobble), and process parameters on bead morphology, mechanical performance, metallurgical features, and electrical resistance was systematically examined. The key findings are summarized as follows:

- Under the investigated line-welding window, defects were frequently observed, highlighting the sensitivity of tab welding to parameter matching and thermal boundary conditions.
- The wobble-based seam strategy produced a wider effective bonded region and improved joint integrity in the foil-stack-top configuration, reaching a maximum tensile–shear load of approximately 1.28 kN at 0.8 mm.
- The same strategy reduced electrical resistance to as low as 45.3 µΩ at 1.2 mm, which is consistent with an increased conductive cross-section and reduced discontinuities in the current path.
- The top lead-tab configuration remained more sensitive to bead instability, which is attributed to thermal boundary conditions and heat extraction pathways; a strictly matched A/B comparison will be addressed in future work.

The results provide a practical process window for a wobble-based seam strategy to achieve mechanically robust and electrically stable tab joints in pouch-cell manufacturing, while highlighting the role of lap configuration through thermal boundary conditions.

## Figures and Tables

**Table 1:** Welding parameters.

Seam Strategy	Line	Wobble
Laser focal position and power	0 mm (top surface), 2000 W	
Welding speed (mm/s)	100, 150, 200 (3 levels)	50
Wobble amplitude (mm)	-	0.4, 0.8, 1.2 (3 levels)
Wobble Frequency (Hz)	-	625, 313, 208 (3 levels)
Overlap (%)	-	80
Beam diameter (µm)	273	
Shielding gas (L/min)	20 (Nitrogen)	

**Table 2:** Chemical composition and mechanical properties of the base metal (Cu lead tab and foil stack).

**Chemical composition (wt. %)**		
Cu	Fe	Mn
Bal.	<1	<0.5
**Mechanical properties**		
Yield strength (MPa)	Tensile strength (MPa)	Elongation (%)
205	260	25

## Data Availability

All authors of this article agreed to share and republish their research data. The original contributions presented in this study are included in the article. Further inquiries can be directed to the corresponding authors.
